# Supplementary material for: Bacteriophage Cocktails in the Post-COVID Rehabilitation
Source: Viruses. 2022 Nov 23;14(12):2614. doi: 10.3390/v14122614 (PMC9783051; doi:10.3390/v14122614)
Supplement: Supplementary file 1 [file viruses-14-02614-s001.zip › viruses-1990878-supplementary/Supplementary files/Table S1.pdf]

Table S1. Clinical and laboratory parameters in the control group. Data are presented as median and interquartile range (IQR).

Parameters	Control group Me (IQR)		p-value	p-value Mann-Whitney test	
	Before n = 8	After n = 8	Wilcoxon test Before VS After	Bph vs control group Before	Bph vs control group After
Saturation, percent	98 (97–98)	97 (97–99)	0,999	0,001*	0,019*
Respiratory rate	17 (16–18)	16 (16–17)	0,102	<0,001*	0,297
Heart rate,	76 (72–85)	77 (70–85)	0,999	0,820	0,050
Bilirubin, µmol/l	99 (7.0–17.2)	10.9 (7.5–14.4)	0.674	0.162	0.254
Total protein,g/l	73 (67.6–75.6)	72,3 (70.7–73.5)	0.327	0.074	0.374
Creatinine, µmol/l	84 (81–92)	88 (80–94)	0.484	0.853	0.789
Glucose, mmol/l	5.0 (4.8–5.3)	5.1 (4.7–5.9)	0.208	0.017*	0.138

Cholesterol, mmol/l	5.36 (4.33–6.00)	4.86 (3.95–5.45)	0.069	0.606	0.204
Lactate dehydrogenase, u/l	165 (154–190)	169 (156–197)	0.327	0.124	0.098
Alanine aminotransferase, u/l	15.2 (13.1–22.7)	16.4 (12.6–24.6)	0.484	0.113	0.150
Aspartate aminotransferase, u/l	20.0 (18.9–22.8)	21.6 (19.1–33.5)	0.123	0.334	0.821
C-reactive protein, mg/l	0.1 (0.1–0.8)	0.75 (0.63–0.88)	0.021*	0.190	0.009*
Total bacterial mass	$4*10^{13}$ ($2*10^{13}$–$1.6*10^{14}$)	$1,5*10^{13}$ ($8*10^{12}$–$2*10^{13}$)	0.035*	0.611	0.237
Lactobacillus spp.	$6*10^{6}$ ($1*10^{5}$–$1*10^{8}$)	$7.5*10^{6}$ ($1*10^{6}$–$6*10^{7}$)	0.575	0.707	0.237
Bifidobacterium spp.	$4.5*10^{10}$ ($9*10^{9}$–$2*10^{11}$)	$1.5*10^{18}$($1.5*10^{9}$–$7*10^{10}$)	0.058	0.411	0.674
Escherichia coli	$3.5*10^{8}$ ($6*10^{7}$–$1*10^{9}$)	$4.5*10^{7}$ ($2.5*10^{6}$–$2*10^{8}$)	0.012*	0.674	0.295
Bacteroides spp.	$4*10^{13}$ ($2*10^{13}$–$1*10^{14}$)	$1.5*10^{13}$ ($8*10^{12}$–$2*10^{13}$)	0.035*	0.611	0.237
Faecalibacterium prausnitzii	$5*10^{11}$ ($4*10^{11}$–$4*10^{12}$)	$1.5*10^{11}$ ($5*10^{10}$–$3*10^{11}$)	0.017*	0.006*	0.947
Klebsiella pneumoniae	-	-	-	-	-
Klebsiella oxytoca	-	-	-	-	-

Candida spp.	$7*10^5$ ($7*10^5$–$7*10^5$)	-	-	0.999	-
Staphylococcus aureus	$4*10^6$ ($3*10^5$–$4*10^6$)	$5*10^6$ ($1*10^6$–$5*10^6$)	0.655	0.262	0.183
Enterococcus spp.	-	-	-	-	-
Bacteroides thetaiotaomicron	$3,4*10^9$ ($8*10^7$–$1.1*10^{10}$)	$2*10^9$ ($1*10^8$–$4*10^9$)	0.173	0.590	0.664
Akkermansia muciniphila	-	$1.5*10^8$ ($1*10^8$–$2*10^8$)	-	-	0.533
Clostridium difficile	-	-	-	-	-
Clostridium perfringens	-	-	-	-	-
Proteus spp.	$2*10^9$ ($2*10^9$–$2*10^9$)	-	-	0.500	-
Enterobacter spp.	$1*10^8$ ($5*10^6$–$2*10^{10}$)	$4*10^7$ ($8*10^6$–$1*10^9$)	0.600	0.078	0.288
Citrobacter spp.	-	-	-	-	-
Fusobacterium nucleatum,	$7.5*10^6$ ($5*10^6$–$7.5*10^6$)	$3*10^5$ ($3*10^5$–$3*10^5$)	-	0.133	0.580
Parvimonas micra	-	-	-	-	-
Ratio *Bacteroides fragilis* group/ *Faecalibacterium prausnitzii*	100 (37–779)	163 (100–200)	1.000	0.172	0.999
hemoglobin, g/L	137 (116;150)	130 (117;154)	0.889	0.452	0.169

White blood cells, 10^9/l	5.4 (5.1–5.8)	5.5 (4.8–5.9)	0.735	0.463	0.514
Erythrocyte Sedimentation Rate (ESR), mm/hour	14.0 (8.0–25.0)	12.5 (6.0–20.3)	0.362	0.563	0.950
