# Supplementary material for: Bacteriophage Cocktails in the Post-COVID Rehabilitation
Source: Viruses. 2022 Nov 23;14(12):2614. doi: 10.3390/v14122614 (PMC9783051; doi:10.3390/v14122614)
Supplement: Supplementary file 1 [file viruses-14-02614-s001.zip › viruses-1990878-supplementary/Supplementary files/Table S2.pdf]

Table S2. CT lung volume assessment in subjects on bacteriophage therapy (BPh).

ID	Retrospective Data	After Bhp (10–14 days)						
	CT scan lung damage (%)	Lung damage (%)	Lung capacity (cm3)	Lung damage (cm3)	Left lung damage (cm3)	Left lung damage (%)	Right lung damage (cm3)	Right lung damage (%)
1	20	9	5148	461	196	8	265	11
2	25	11	3759	400	226	13	174	9
3	5	1	4276	60	32	2	29	1
4	10	1	4668	40	16	1	24	1
5	40	2	6246	101	53	2	54	2
6	10	2	6283	142	63	2	79	3
7	20	1	4609	47	19	1	27	1
8	80	30	2700	783	418	33	366	26
9	40	1	6153	59	25	1	34	1
10	25	6	3229	179	62	4	117	7
11	44	2	6130	138	63	2	76	2
12	60	14	3531	494	209	13	285	15
13	25	8	4702	390	265	13	125	5
14	75	42	3624	1475	703	43	772	41
15	25	4	3607	126	59	4	67	4
16	20	1	4512	34	20	1	14	1
17	15	1	8016	117	65	1	52	1
18	10	3	6979	232	80	2	153	5
19	15	5	6535	303	121	4	183	6
20	68	26%	3220	800	436	22	364	31
