# Supplementary figures and images for: Bacteriophage Cocktails in the Post-COVID Rehabilitation
Source: Viruses. 2022 Nov 23;14(12):2614. doi: 10.3390/v14122614 (PMC9783051; doi:10.3390/v14122614)

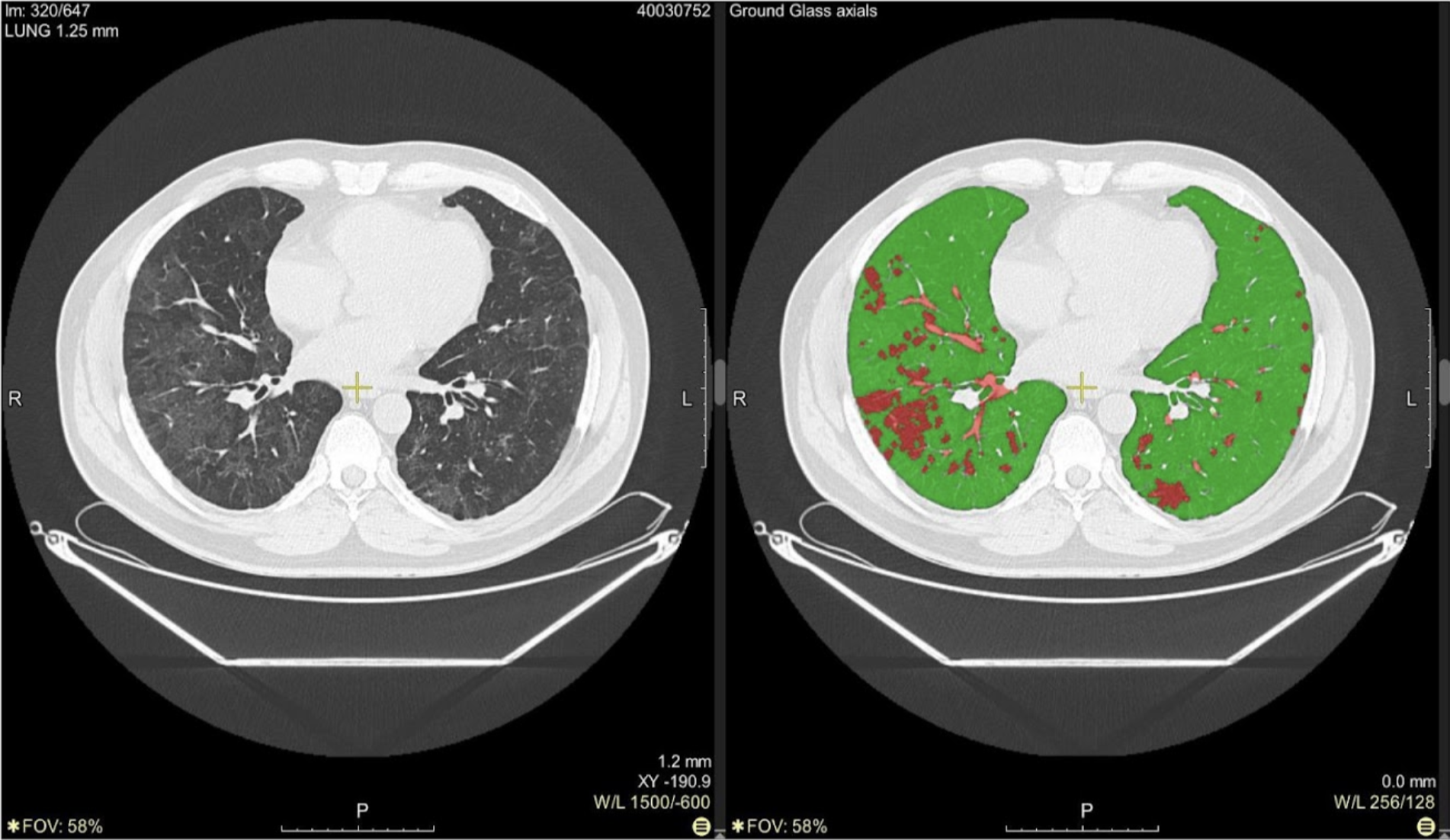

Supplement: Supplementary file 1 [file viruses-14-02614-s001.zip › viruses-1990878-supplementary/Supplementary files/Figure S1.png]
